# Adjusting to self in the thymus: CD4 versus CD8 lineage commitment and regulatory T cell development

**DOI:** 10.1084/jem.20230896

**Published:** 2024-07-09

**Authors:** Isabel Baldwin, Ellen A. Robey

**Affiliations:** 1 Division of Immunology and Molecular Medicine, Department of Molecular and Cell Biology, University of California, Berkeley, Berkeley, CA, USA

## Abstract

During thymic development, thymocytes adjust their TCR response based on the strength of their reactivity to self-peptide MHC complexes. This tuning process allows thymocytes with a range of self-reactivities to survive positive selection and contribute to a diverse T cell pool. In this review, we will discuss recent advances in our understanding of how thymocytes tune their responsiveness during positive selection, and we present a “sequential selection” model to explain how MHC specificity influences lineage choice. We also discuss recent evidence for cell type diversity in the medulla and discuss how this heterogeneity may contribute to medullary niches for negative selection and regulatory T cell development.

## Teaser

This review covers recent advances in our understanding of how developing T cells sense and adjust to their self-reactivity and how this impacts their decision to become a CD8 T cell, a CD4 T cell, or a regulatory T cell.

## Introduction

From the time that developing thymocytes first express their newly formed αβTCRs to the time they emerge from the thymus to enter circulation as mature T cells, they undergo a stringent selection process that shapes the mature αβTCR repertoire. This selection process includes both negative selection, which prevents autoimmunity by eliminating most strongly self-reactive T cells, as well as positive selection, which ensures that surviving T cells express a functional TCR that has the appropriate affinity for the individual’s MHC proteins. In the traditional view, thymic selection was primarily about survival: whether a thymocyte could avoid death by neglect and death by negative selection to become a mature T cell. However, more recently that view has shifted to one that recognizes that all mature T cells have some capacity to recognize self and that the extent of that self-reactivity is a key determinant in shaping T cell functional capacity during thymic selection. In this new view, thymic selection is an active process, in which T cell responsiveness and functionality are “tuned” to match each T cell’s self-reactivity. This process leads to conventional CD4 and CD8 T cells that can fill different functional roles during immune responses (Cho and Sprent, 2018; Hogquist and Jameson, 2014; Klein et al., 2014; This et al., 2023) and to regulatory T cells (Treg) that use their self-reactivity to keep conventional autoreactive T cells in check (Klein et al., 2019; Owen et al., 2019b; Savage et al., 2020).

**Figure 1.**
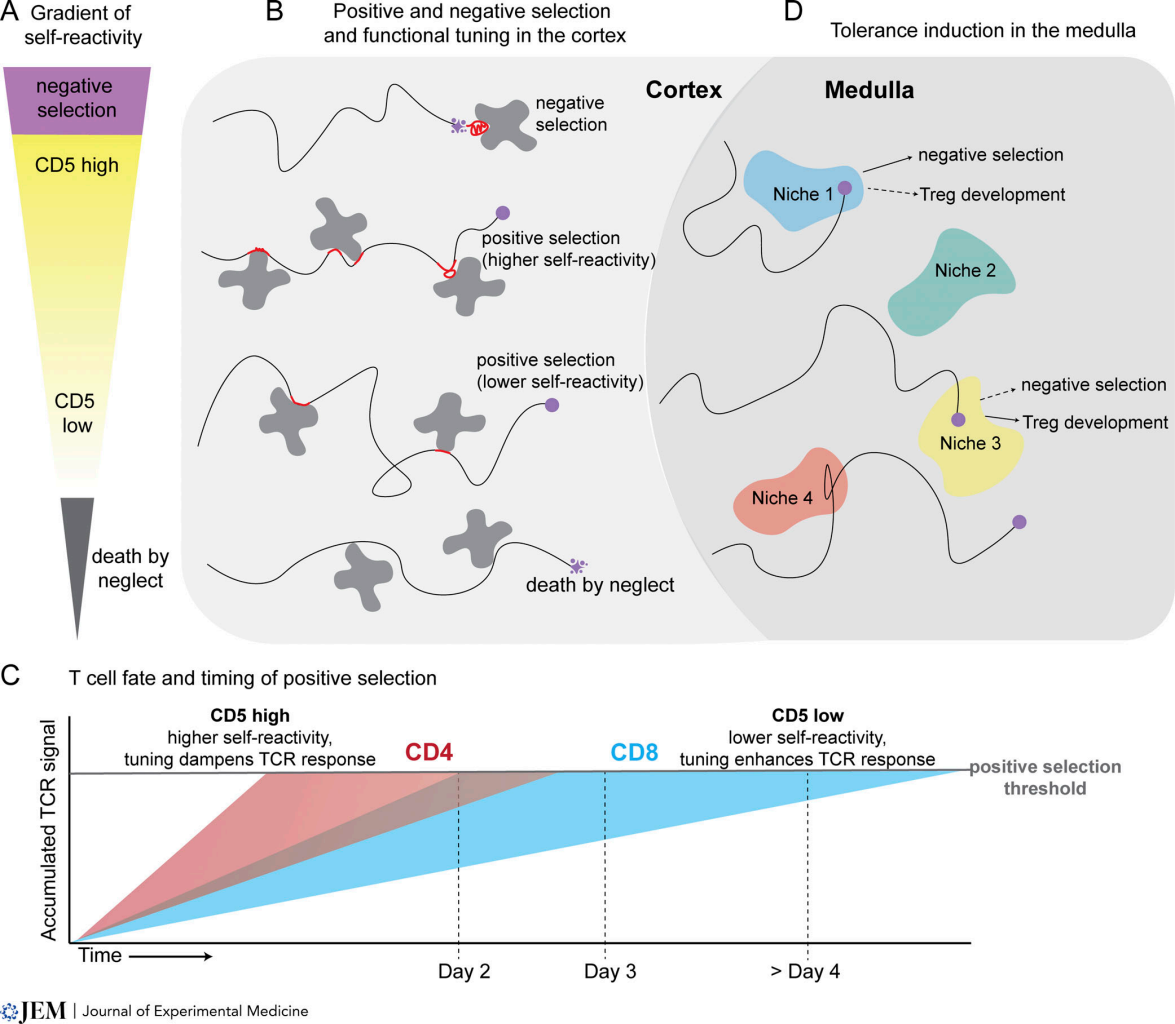
**Thymic selection and functional tuning. (A)** Thymocyte fate in the cortex is linked to self-reactivity, with strong self-reactivity leading to negative selection (purple; also called clonal deletion) and very low self-reactivity leading to a failure of positive selection (gray; also called death by neglect). Within the moderate range of self-reactivity that allows for positive selection, thymocytes at the higher end downmodulate their TCR responsiveness, correlating with increased expression of CD5, whereas those with the lowest self-reactivity upregulate their responsiveness, correlating with lower expression of CD5. For mature peripheral T cells, differences in self-reactivity (as read out by CD5 levels) correlate with their functional potential. See text for details. **(B)** During the initial phase of thymic selection, thymocytes migrate through the cortex while testing their newly formed TCR for self-recognition. During negative selection thymocytes undergo migratory arrest and experience persistent TCR signals (red portion of the migration path) and die after a few hours. Thymocytes with more moderate reactivity experience transient TCR signals during migratory pauses, with the duration of signaling events correlating with the degree of self-reactivity. **(C)** T cell fate correlates with the time required to assess self-reactivity and complete positive selection. Note that very rapid accumulation of TCR signals leads to negative selection of both MHC I– and MHC II–specific thymocytes (not depicted). **(D)** After undergoing positive and negative selection in the cortex, thymocytes undergo screening for tolerance to diverse tissue-restricted antigens displayed in a mosaic pattern in the thymic medulla. Whether self-reactive CD4 T cells undergo negative selection, develop as Tregs, or remain as conventional CD4 T cells depends on both on the strength of their reactivity to tissue-restricted antigens, and their ability to access limiting niches containing antigen-bearing APCs, IL2, and other cytokines.

The T cell surface protein CD5 has been key to our understanding of T cell functional tuning. CD5 expression is upregulated by TCR signaling during positive selection and also serves as a negative regulator of TCR, thereby contributing to the tuning process (Azzam et al., 1998, 2001). Moreover, the levels of CD5 on naïve T cells serve as a reliable surrogate marker for self-reactivity, facilitating functional comparisons of T cells with relatively high versus low self-reactivity (Cho et al., 2016; Fulton et al., 2015; Mandl et al., 2013; Persaud et al., 2014; Zinzow-Kramer et al., 2019) (Fig. 1 A). CD5^high^ (more self-reactive) T cells show more rapid expansion following antigen encounter and exhibit greater cytokine responsiveness compared with CD5^low^ cells. On the other hand, there is evidence that CD5^low^ CD8 T cells are more sensitive to TCR triggering (Cho et al., 2016), can survive better without IL7 (Palmer et al., 2011), and are more resistant to exhaustion during chronic infections (Tsitsiklis et al., 2020). CD5^low^ CD4 T cells appear to be more resistant to apoptosis (Weber et al., 2012). Thus, T cells with high and low self-reactivity may serve different purposes during immune responses.

CD5 expression levels and the correlated functional differences are in large part imprinted in the thymus (Persaud et al., 2014). Thymocyte functional tuning correlates with the dynamics of TCR signaling during thymic development (Fig. 1 B). Long lasting contacts with APCs and persistent TCR signals lead to cell death (negative selection), while transient contacts and intermittent signals allow thymocytes to avoid death while accumulating TCR signals and tuning their responsiveness (Au-Yeung et al., 2014; Dzhagalov et al., 2013; Kurd and Robey, 2016; Melichar et al., 2013, 2015) (Fig. 1 A). Interestingly, CD4 single positive (SP) thymocytes, which are inherently more self-reactive than CD8 SP thymocytes (Moran et al., 2011: see Box 1), complete positive selection after 1–2 days, compared to ≥3 days for CD8 SP (Lucas et al., 1993; Lutes et al., 2021; Saini et al., 2010). Moreover, MHC-I–specific thymocytes of particularly low self-reactivity can take a week or longer to complete positive selection, and this correlates with very brief and infrequent TCR signals (Lutes et al., 2021) (Fig. 1, B and C). These observations suggest that the rate of TCR signal accumulation, dictated by the strength of self-recognition, determines whether T cells undergo negative selection or become CD4 or CD8 T cells, as well as determines functional tuning within the CD4 and CD8 lineages (Fig. 1 C). It is noteworthy that tuning based on self-reactivity does not uniformly dampen all TCR responses. If it did, tuning would wipe out functional differences due to self-reactivity and all T cells would be functionally equivalent. Instead, tuning selectively dampens some responses, while leaving others intact, allowing thymocytes to avoid negative selection while also introducing functional heterogeneity.

Box 1 Two labs developed transgenic mouse strains expressing fluorescent TCR signaling reporters by placing GFP coding regions under the control of the TCR target gene Nur77 (Moran et al., 2011; Zikherman et al., 2012). The Nur77-GFP reporter mice were used to demonstrate that CD4 naïve T cells are more self-reactive than naïve CD8 T cells and that Tregs are more self-reactive than conventional CD4 T cells.

Thymocytes that survive positive selection and the first round of negative selection in the thymic cortex, then migrate to the medulla where they encounter a diverse set of tissue-restricted self-antigens and undergo a second round of tolerance screening (Fig. 1 D). While some thymocytes whose TCRs recognize medullary self-antigens undergo negative selection, others undergo an additional round of functional tuning at this stage by upregulating the transcription factor FoxP3 and adopting an immunosuppressive Treg fate. In the medulla, the fate of self-reactive T cells depends on multiple factors. First, newly positively selected CD4 SP thymocytes are more prone to undergo deletion (Hogquist et al., 2015) and Treg development (Wirnsberger et al., 2009) upon encounter with high-affinity self-ligand compared with more mature CD4 SP. Medullary microenvironments also play an important role in determining T cell fate. Self-reactive T cells are subjected to competition for limiting niches that contain rare APCs presenting tissue-restricted self-antigens as well as limiting amounts of IL2 and related cytokines. A self-reactive CD4’s ability to compete for resources in the niche can determine whether they are negatively selected or develop into Tregs or leave the thymus as autoreactive conventional T cells (Klein et al., 2019; Owen et al., 2019b; Savage et al., 2020).

The past few years have seen important advances in our understanding of T cell selection in the thymus. These include single-cell, multiomic analyses that provide a detailed timeline of gene expression changes during positive selection (Steier et al., 2023) and mouse genetic experiments that demonstrate the key role of CD4 and CD8 coreceptor expression dynamics in lineage commitment (Shinzawa et al., 2022). Regarding Treg development, recent studies have revealed the heterogeneity of medullary thymic epithelial cells (mTECs) (Bornstein et al., 2018; Givony et al., 2023; Michelson et al., 2022; Michelson and Mathis, 2022; Miller et al., 2018) and the composition of medullary niches that contribute to self-tolerance by driving Treg cell development and negative selection. In this review, we will discuss how recent advances have shaped our understanding of the processes of positive selection and tolerance mechanisms, with an emphasis on how self-reactivity shapes T cell fate and functionality.

## Adjusting to self in the cortex: Positive selection and lineage commitment

### Thymocytes modulate their TCR sensitivity during positive selection

Over two decades ago, two groups reported that CD4 CD8 double positive (DP) thymocytes that had not yet initiated positive selection (called preselection DP) were highly sensitive to low-affinity ligands, despite their relatively low level of surface TCR (Davey et al., 1998; Lucas et al., 1999). This surprising property of preselection DP was proposed to endow thymocytes with the ability to recognize and respond to the relatively weak self-peptide-MHC ligands that drive positive selection, while the gradual loss of sensitivity as thymocytes mature helps them to avoid overt self-reactivity in the periphery. Since then, information has been accumulating about how TCR responsiveness is modulated during positive selection.

As discussed above, CD5 is an important player in modulating TCR responses. CD5 expression is low on preselection DP and is induced during the initial phase of positive selection. In addition, CD5 downmodulates TCR signaling, providing negative feedback (Azzam et al., 1998, 2001). Dynamic regulation of CD5 expression at later stages of positive selection may also contribute to CD4 versus CD8 lineage commitment, as discussed below.

The enigmatic signaling molecule THEMIS is preferentially expressed in DP thymocytes, modulates TCR signaling, and is required for efficient positive selection (Fu et al., 2009; Johnson et al., 2009; Kakugawa et al., 2009; Lesourne et al., 2009; Patrick et al., 2009). THEMIS associates with the TCR signaling complex and regulates its activity, at least in part via interactions with the tyrosine phosphatase, SHP1. In some studies, THEMIS has been shown to enhance SHP1 function and thereby dampen TCR signaling (Fu et al., 2013, 2014). In contrast, other studies provided evidence that THEMIS negatively regulates SHP1, thereby enhancing TCR responses (Choi et al., 2017a, 2017b; Zvezdova et al., 2016). The complexities of THEMIS function are highlighted by a recent study showing that a single tyrosine in THEMIS is phosphorylated by the TCR-associated src family tyrosine kinase LCK and dephosphorylated by SHP1 and that THEMIS has both phosphorylation-dependent and independent functions (Zhang et al., 2024). These seemingly contradictory results can be reconciled by the proposal that THEMIS is a bidirectional TCR tuning molecule. THEMIS would inhibit SHP1 during the initial response to positive selecting ligands, thereby enhancing TCR responses. Once TCR signaling begins, LCK phosphorylation would allow THEMIS to activate SHP1, leading to negative feedback of the TCR signal. In this way, bidirectional tuning by THEMIS could contribute to the transient TCR signals that occur during positive selection, allowing thymocytes to slowly accumulate TCR signals while avoiding a rapid rise in TCR signals that could lead to negative selection (Fig. 1, B and C).

Other mysterious players that contribute to the TCR sensitivity of preselection DP thymocytes are voltage-gated ion channels. The regulatory subunit of a voltage-gated sodium channel, *Sna4b*, is selectively expressed in DP thymocytes and is required for their sustained calcium flux in response to positive selecting ligands and for efficient CD4 SP development (Lo et al., 2012). Intriguingly, MHC-I–specific thymocytes with particularly low self-reactivity retain the expression of *Sna4b* as well as components of a voltage-gated calcium channel (*CacnA1e* and *CacnB3*) as they undergo positive selection (Lutes et al., 2021). These ion channels are best known for their roles in propagating action potentials in neurons, and it is unclear how they help to enhance TCR signals in thymocytes, which are not electrically excitable. One intriguing possibility is that these channels mediate more subtle changes in membrane polarity, which may enhance weak TCR signals by facilitating calcium entry and/or by altering TCR conformation (Feske et al., 2015; Trebak and Kinet, 2019).

### TCR tuning and lineage commitment

As thymocytes undergo positive selection and TCR tuning based on their self-reactivity, they are also choosing between the CD4 and CD8 T cell fates. This is a lengthy process, requiring ∼2 days for CD4 SP and up to 2 wk for CD8 SP to emerge after their initial encounter with their positive selecting ligands (Kurd and Robey, 2016; Lucas et al., 1993; Lutes et al., 2021; Saini et al., 2010). During that time, thymocytes undergoing positive selection experience changes in the expression of CD4 and CD8 co-receptors, as well as other molecules that modulate TCR responsiveness. TCR signaling dynamics are key to accurately link CD4 or CD8 expression, and the corresponding helper or killer effector functions, to MHC-II or MHC-I recognition.

Although positive selection and lineage commitment have been intensely studied for decades, two recent studies have provided a new perspective on the key events that link MHC recognition to T cell fate. One is an elegant genetic study in which the CD4 coding regions were engineered into the CD8 gene locus and vice versa (Shinzawa et al., 2022) (Box 2). In this strain, termed FlipFlop mice, the reversal of coreceptor expression patterns led to a striking lineage reversal, in which thymocytes bearing MHC-II–specific TCRs gave rise to cytotoxic lineage CD4^+^ T cells, and those with MHC-I–specific TCR gave rise to helper lineage CD8^+^ T cells. While previous studies reported some lineage redirection in mice with altered co-receptor expression patterns (Davis et al., 1993; Itano et al., 1994; Matechak et al., 1996), the relatively efficient lineage redirection observed in FlipFlop mice clearly illustrated the key driving role of co-receptor expression on the lineage choice.

Box 2 In FlipFlop mice (Shinzawa et al., 2022), the coding regions of CD4 are inserted in the *Cd8* locus, replacing the endogenous CD8 coding sequences, and the coding regions of CD8α and CD8β are inserted into the *Cd4* locus, replacing the endogenous CD4 coding sequence. In these mice, thymocytes undergo lineage reversal; those bearing MHC-II–specific TCRs develop into “cytotoxic” lineage cells, as indicated by expression of the CD8-defining transcription factor *Runx3*, whereas those bearing MHC-I–specific TCRs develop in the “helper” lineage as indicated by expression of the CD4 defining transcription factor *Thpok/Zbtb7b*.

In another advance, we and our collaborators used a multiomics approach to provide a detailed timeline of the mRNA and protein expression changes that accompany positive selection of CD4 or CD8 fated cells (Steier et al., 2023). In this section, we will summarize our current understanding of the timeline of key events during positive selection and lineage commitment and frame these events in terms of a Sequential Selection model (Steier et al., 2024). This model helps to explain the link between MHC recognition and CD4 versus CD8 lineage commitment and highlights the importance of TCR signal modulation throughout positive selection.

**Figure 2.**
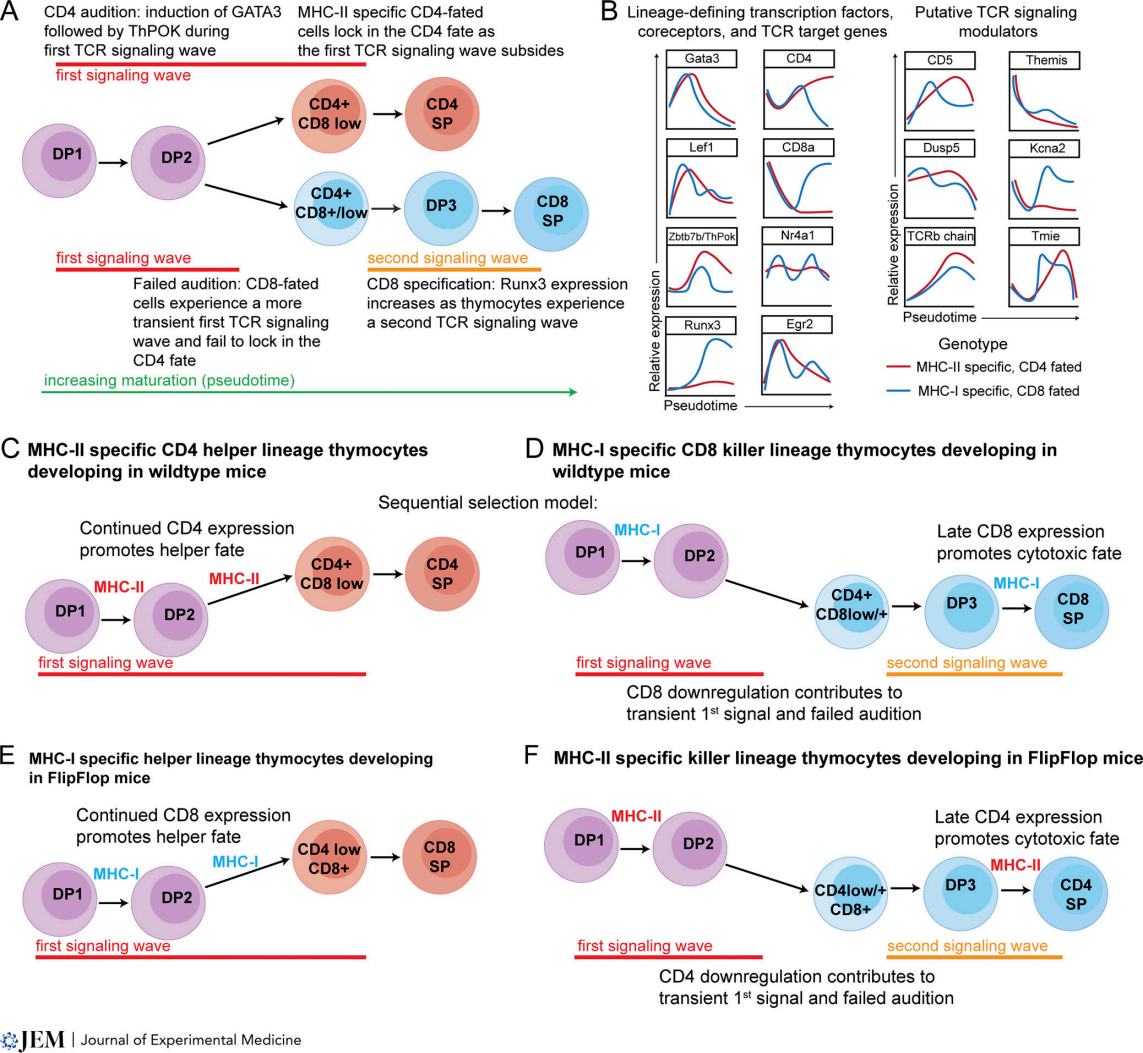
**A sequential selection model for CD4/CD8 lineage commitment. (A)** A timeline of gene expression changes during positive selection based on CITEseq analyses of thymocytes from lineage-restricted mice (Steier et al., 2023). During the initial phase of positive selection, both CD4-fated (MHC-II–specific) and CD8-fated (MHC-I–specific) thymocytes undergo a “CD4 audition” in which upregulation of the transcription factor GATA3 is followed by upregulation of the CD4-defining transcription factor ThPOK and downregulation of CD8 during an initial wave of TCR signaling (red lines). CD8-fated thymocytes experience a more transient first signaling wave and a failed CD4 audition, and later go on to upregulate the CD8-defining transcription factor RUNX3 and undergo a second TCR signaling wave (orange line). See text for details. **(B)** Idealized expression versus pseudotime plots of selected genes. Red lines represent data from thymocytes from three different CD4-fated mouse strains (AND and OTII TCR transgenic and MHC-I deficient [b2-microglobulin ko]) and blue lines represent data from three different CD8-fated mouse strains (F5 and OTI TCR transgenic and MHC-II deficient [IA^b^ ko]). All plots refer to mRNA expression, except for CD4, CD8, CD5, and TCRb which refer to protein data based on DNA-barcoded antibodies. The original data can be found in supplementary information from Steier et. al. (2023). **(C–F)** A sequential selection model accounts for the link between recognition of MHC-I versus MHC-II and CD4 versus CD8 T cell fate. **(C)** In wild-type mice, thymocytes undergoing selection on MHC-II (fated for the CD4/helper lineage) experience a prolonged first signaling wave (red line) as they downregulate CD8 and maintain CD4 expression allowing them to fully upregulate GATA3 and lock in expression of THPOK. **(D)** Thymocytes undergoing selection on MHC-I (fated for the CD8/cytotoxic lineage) experience a more transient signaling wave, in part due to the downregulation of CD8 and the resulting loss of MHC-I recognition during the CD4 audition phase. The second signaling wave in CD8-fated cells serves as a check to ensure a match between the MHC-I specificity of the TCR and the expression of the corresponding coreceptor CD8. Any MHC-II–specific thymocytes that failed the CD4 audition (perhaps due to weak self-reactivity) would be eliminated at this stage since they would not have the appropriate match between co-receptor expression and MHC recognition. **(E)** In FlipFlop mice, in which CD4 and CD8 expression patterns are reversed (Shinzawa et al., 2022), some MHC-I–specific thymocytes can adopt the helper fate during the “helper audition” due to prolonged expression of CD8 during the initial signaling wave. **(F)** MHC-II–specific thymocytes in FlipFlop mice fail the “helper audition” due to downregulation of CD4 and the subsequent decline in TCR signal. Later, they can adopt the cytotoxic fate as they experience a second TCR signaling wave due to late expression of CD4.

To construct a timeline of positive selection and lineage commitment, we and our collaborators used cellular indexing of transcriptomes and epitopes (CITEseq; see Box 3) to measure the transcriptome and cell surface proteome of individual thymocytes from wild-type and lineage-restricted (TCR transgenic and MHC-I– or -II–deficient) mice. We then used these data to infer a relative ordering of coreceptor, lineage-defining transcription factors, and TCR signaling target gene expression along a developmental trajectory (Steier et al., 2023) (Fig. 2, A and B). While many of the events were predicted based on earlier studies, two unexpected features stood out. One is the strikingly parallel expression of CD4-lineage–defining transcription factors: *Gata3* followed by *Thpok* and accompanied by CD8 downregulation in both CD4- and CD8-fated cells (Steier et al., 2023) (Fig. 2 B). Thus, the initial phase of positive selection can be thought of as an “audition” for the CD4 fate. A second unexpected observation is the presence of a distinct second wave of TCR target gene expression (e.g., *Egr2* and *Nur77*; Fig. 2, A and B) in CD8-fated cells after the CD4 audition and overlapping with induction of the CD8-defining transcription factor *Runx3*. This late, CD8-specific TCR signaling wave indicated that the most prominent model for lineage commitment, termed kinetic signaling (Singer et al., 2008), may require revision. In this model, the sole function posited for TCR signaling is to drive the CD4 fate during the initial phase of positive selection. Indeed, the kinetic signaling model invokes an interruption of TCR signaling as a key driver of the CD8 fate, with no mechanism for testing for a match between CD8 expression and MHC-I recognition in CD8-fated cells.

Box 3 CITEseq is a method to measure the transcriptome and cell surface proteome of individual cells (Stoeckius et al., 2017). It combines single-cell RNA sequencing with a panel of antibodies labeled with unique oligonucleotide barcodes. Antibody binding and RNA transcripts can then be read out together, allowing for the integration of gene and protein expression using computational tools such as TotalVI (Gayoso et al., 2021). Furthermore, developmental trajectories in the data can be inferred using pseudotime analysis tools such as SlingShot (Street et al., 2018).

We recently proposed a revised model called sequential selection based on our current understanding of the events accompanying positive selection (Steier et al., 2024) (Fig. 2, C and D). This model incorporates some aspects of kinetic signaling, as well as earlier models termed stochastic/selection and instruction (Germain, 2002; Robey and Fowlkes, 1994; Steier et al., 2024). It emphasizes the importance of TCR signaling after co-receptor downregulation in both lineages as a test for an appropriate match between TCR specificity for MHC-I versus MHC-II and the CD8/killer versus CD4/helper T cell fates. Our model aligns well with the results from co-receptor reversed FlipFlop mice (Shinzawa et al., 2022) (Fig. 2, D and E). Lineage reversal of MHC-I–specific thymocytes would result from the sustained expression of CD8 during the “helper audition,” allowing some MHC-I–specific thymocytes to complete the audition (Fig. 2 E). In addition, lineage reversal of MHC-II–specific thymocytes to the cytotoxic fate would result both from a loss of signal during the initial phase of positive selection leading to a failed helper lineage audition and the ability to undergo a second wave of TCR signaling as CD4 expression recovers and CD8 expression declines (Fig. 2 F).

In addition to invoking a late TCR-driven step in CD8 lineage specification, there are other important differences between the sequential selection and kinetic signaling models. The kinetic signaling model invokes CD8 downregulation as an obligatory step in driving the CD8 fate. In contrast, the sequential selection model posits that any thymocyte with relatively weak self-reactivity would fail to adopt the CD4 fate simply because they cannot accumulate sufficient signal during the limited developmental window of the CD4 audition. According to the sequential selection model, CD8 downregulation provides a fail-safe mechanism to ensure that MHC-I–specific thymocytes with particularly high self-reactivity do not complete the CD4 audition. This is consistent with the observation that MHC-I–specific thymocytes can efficiently give rise to CD8 SP thymocytes even in transgenic mice with constitutive CD8 expression (Itano et al., 1994; Kojo et al., 2020). Moreover, while kinetic signaling posits that a sustained TCR signal is sufficient to ensure that MHC-II–specific thymocytes adopt the CD4 fate, the sequential selection model predicts that MHC-II–specific thymocytes with relatively low self-reactivity would fail the CD4 audition but be prevented from adopting the CD8 fate when the loss of CD4 expression prevents them from sustaining a second signaling wave. This is consistent with the observation that some MHC-II–specific thymocytes can give rise to CD8 lineage T cells in transgenic mice with constitutive CD4 expression (Davis et al., 1993).

Another key difference between the models is that kinetic signaling exclusively invokes co-receptor expression to modulate TCR signals during lineage commitment, whereas sequential selection posits that other modulators of TCR signaling contribute to the dynamic pattern of TCR signaling during positive selection, and therefore help to guide the lineage choice. As discussed earlier, preselection thymocytes gradually reduce their TCR sensitivity as they initiate positive selection, in part by the downregulation of signal-enhancing factors such as ion channels and THEMIS, and by the upregulation of signal-dampening factors such as CD5 (Hogquist and Jameson, 2014) (Fig. 2 B). In addition, the initial TCR signal activates negative feedback by dual phosphatases DUSP2 and 5, which are induced by TCR signals and then act to turn down the MEK/ERK branch of the TCR signaling pathway (Bettini and Kersh, 2007; Kovanen et al., 2008). The gradual loss of TCR signals during the initial phase of positive selection may impose a time limit on completing the CD4 audition. Would-be CD4 T cells would have to accumulate sufficient signal before these negative regulators take full effect. The inherently stronger TCR signals experienced by MHC-II compared with MHC-I–specific T cells (Moran et al., 2011, Box 1), perhaps due in part to the differential usage of LCK by the CD4 versus CD8 cytoplasmic tails (Horkova et al., 2023), would allow MHC-II–specific thymocytes to more efficiently complete the CD4 audition. In line with this idea, the redirection of MHC-I–specific thymocytes to the helper lineage is relatively inefficient in FlipFlop mice, likely due to the weaker signals they experience compared with MHC-II–specific thymocytes in wild-type mice.

After a failed CD4 audition, CD8-fated thymocytes experience a rise in TCR signaling driven by the increase in CD8, as well as other gene expression changes. Indeed, previous studies implicated TCR signal enhancement due to a late rise in expression of the TCR-associated tyrosine kinase Zap70 (Saini et al., 2010), and a loss of CD5 (Chan et al., 1999) as contributing to the development of CD8 SP thymocytes. CITEseq data identified several additional potential TCR regulators that are preferentially expressed in CD8-fated versus CD4-fated cells at this stage, and which may also help to promote the second wave of TCR signaling in CD8-fated thymocytes. These include *Themis*, whose expression is selectively retained as CD8 fated cells mature (Fig. 2 B), and which has been shown to enhance TCR responses in mature thymocytes (Brzostek et al., 2020). In addition, the ion channels *Kcna2* and *Tmie*, are selectively upregulated in CD8-fated cells (Fig. 2 B) and have been implicated in enhancing TCR signals in CD8 T cells with low self-reactivity (Lutes et al., 2021).

It is interesting to consider that the distinct TCR signaling dynamics experienced by CD4 or CD8-fated thymocytes during positive selection could impact their functionality after they leave the thymus. Indeed, naïve CD4 and CD8 T cells respond differently to initial TCR stimulation, with naïve CD8 T cells requiring a briefer initial period of stimulation and dividing more upon priming compared to CD4 T cells (Foulds et al., 2002; Seder and Ahmed, 2003). CD8 cells also proliferate more than CD4 T cells during homeostatic expansion (Guimond et al., 2009). Some of this difference may be due to the co-receptors themselves, given the differential utilization of the src family tyrosine kinase LCK by CD4 and CD8 (Horkova et al., 2023). Developmentally programmed differences in gene expression imposed during positive may also play a role (Fig. 2 B). Most prominently, CD5, which modulates TCR signaling and whose expression varies according to self-reactivity amongst naïve CD4 and CD8 T cells, is markedly higher in CD4 compared with CD8 T cells. In addition, other TCR signal-related molecules are differentially expressed including TCR itself, which is expressed at higher cell surface levels on CD4 compared with CD8 T cells. In summary, becoming a CD4 or CD8 cell is not just about choosing the appropriate co-receptor to match TCR specificity but may also involve long-lasting functional tuning leading to intrinsic differences in TCR responsiveness of these two T cell subsets.

## Adjusting to self in the medulla: Treg development

### Agonist self-ligand and IL2 define limiting niches for thymic T reg development

After selection in the cortex, during which thymocytes adapt to ubiquitous self-antigen by undergoing clonal deletion or functional tuning, thymocytes then undergo a second round of selection in the medulla in response to a wide variety of tissue-restricted antigens. During this second phase of selection, medullary thymocytes may undergo a second round of clonal deletion or may further adapt to strong self-reactivity by upregulating *Foxp3* and adopting a Treg fate. While Tregs can be generated in the periphery from conventional CD4 T cells (Apostolou and Von Boehmer, 2004; Curotto De Lafaille et al., 2004; Knoechel et al., 2005; Kretschmer et al., 2005; Lathrop et al., 2011; Nutsch et al., 2016), the thymus is thought to be the most important site for Treg development (Lee et al., 2011; Savage et al., 2020). Early studies with transgenic mice expressing defined TCRs and their agonist ligands indicated that strong TCR signals could promote Treg development (Apostolou et al., 2002; Jordan et al., 2001; Kawahata et al., 2002). This was corroborated using a TCR signal strength reporter (Nur77-GFP: Box 1), which showed greater expression in Tregs compared with conventional CD4 T cells. On the other hand, the amount of Treg development did not display the expected relationship with the expression levels of the agonist ligand, arguing against a simple instructive role for TCR signals in driving Treg development (Van Santen et al., 2004). A key breakthrough in our understanding of the role of TCR in thymic Treg development came from a quantitative analysis of the TCR repertoire of Tregs compared with conventional CD4 T cells. Sequencing TCRα chains from mice with limited TCR diversity (Box 4) revealed that Tregs and conventional T cells have distinct but partially overlapping TCR repertoires (Hsieh et al., 2006; Lio and Hsieh, 2008). These data supported an instructive role for TCR in Treg development. However, the overlap in repertoire observed in this and another study (Pacholczyk et al., 2007) provided evidence for a large probabilistic component to whether a particular thymocyte would give rise to a Treg or a conventional CD4 T cell.

Box 4 Because the αβTCR repertoire of thymocytes is so diverse, it is challenging to quantitatively analyze the repertoire by sequencing TCRα and β chains from wild-type mice. To get around this problem, several groups have used transgenic mice expressing a fixed TCRβ chain and a heterozygous mutation in TCRα (Hsieh et al., 2004; Malchow et al., 2013; Sant’Angelo et al., 1998). In these mice, there remains sufficient diversity to generate functional T cells, but the diversity is sufficiently limited that the same TCRα sequences can be identified in multiple samples upon deep sequencing. This allows for robust quantitative comparison of the TCR repertoire in different samples (e.g., Treg versus conventional CD4 T cells). Limited TCR diversity mice have been a particularly important tool for the study of Treg development since many manipulations that significantly impact Treg development led to repertoire changes, but not to differences in the overall numbers of thymic Tregs. This likely reflects the competitive nature of thymic Treg development, which tends to normalize the size of the thymic Treg pool based on available IL2 (Weist et al., 2015).

**Figure 3.**
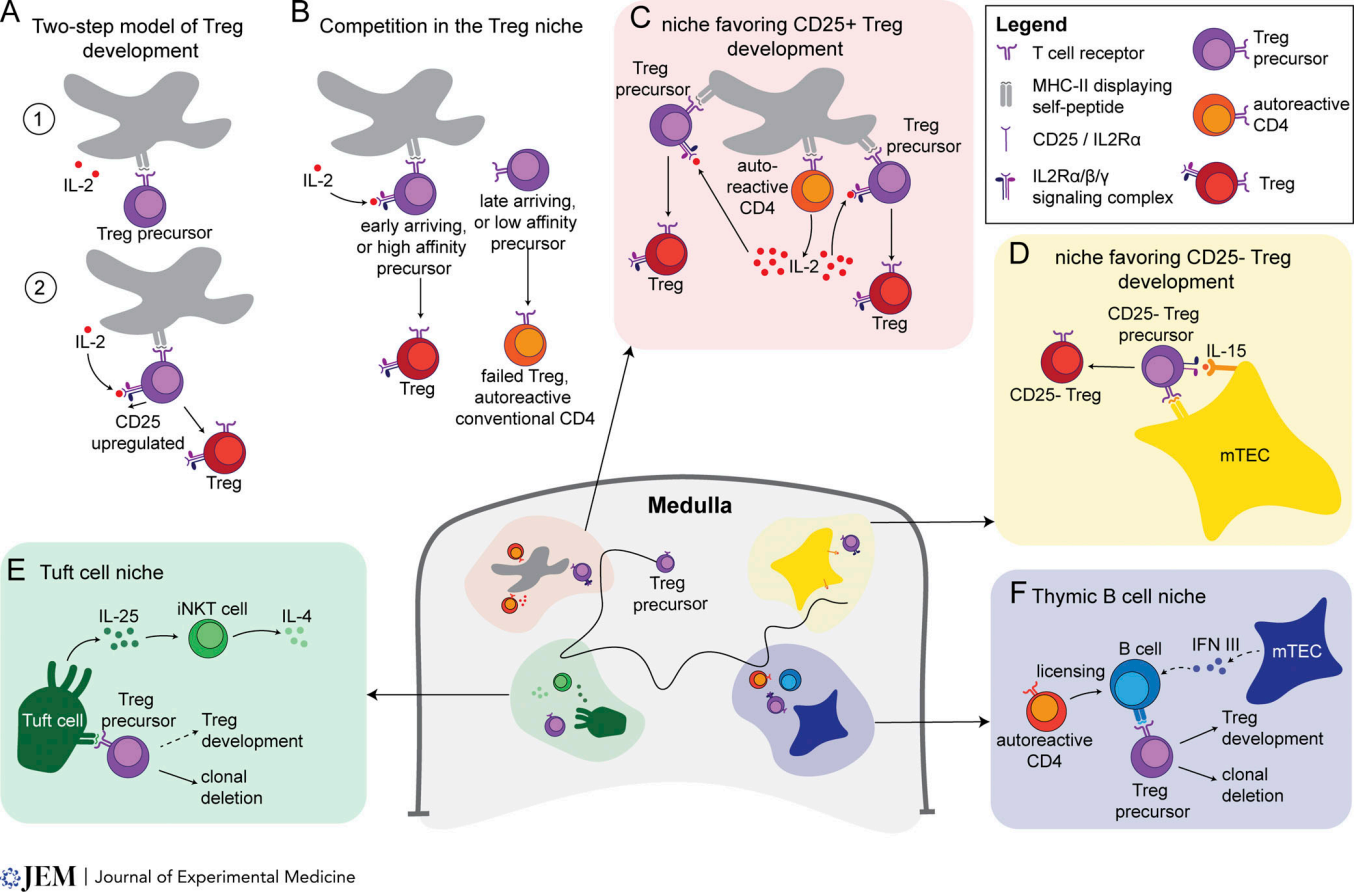
**Diverse medullary niches influence the fate of self-reactive CD4 T cells. (A)** Two-step model of thymic Treg development (Burchill et al., 2008; Lio and Hsieh, 2008). First, a Treg precursor (a newly positively selected CD4 SP thymocyte) receives a strong TCR signal upon recognition of self-peptide presented by a medullary APC. TCR signaling then induces upregulation of CD25, conferring high-affinity IL2 binding. In the second step, IL2 signaling activates STAT5 (not shown) and initiates the expression of the Treg-defining transcription factor, *Foxp3*. **(B)** Competition in the Treg niche. Two Treg precursors with related TCR specificity enter the niche, but one receives a stronger TCR signal because it arrived first to the niche or has a TCR with higher affinity for the self-ligand. The successful precursor then upregulates CD25 and is a better competitor for the local sources of IL2 and can therefore activate STAT5, upregulate *Foxp3*, and develop into a Treg. The other precursor fails to become a Treg and may undergo negative selection or may eventually leave the thymus as an autoreactive conventional CD4 T cell. **(C)** A speculative niche for CD25^+^ Treg development. An autoreactive CD4 SP thymocyte produces IL2 in response to an encounter with high affinity self-ligand. The resulting increase in the amount of local IL-2 allows for multiple Treg precursors with related specificity to successfully develop into Tregs. **(D)** A speculative niche for CD25^−^ Treg development. Treg precursors that directly recognize self-antigens presented by mTECs may rely on IL15 presented by the mTEC to activate STAT5, induce *Foxp3* expression, and develop into Tregs. **(E)** Tuft cell niche. Thymic Tuft cells producing IL25 promote IL4 production by iNKT cells. Together, Tuft cells and iNKT cells provide a localized type 2 cytokine–rich area in the medulla. In this niche, clonal deletion might be favored due to type 2 cytokine–dependent DCs (Breed et al., 2022) (not depicted). **(F)** Thymic B cell niche. In this niche, mTECs provide IFN III and autoreactive CD4s provide CD40L to license thymic B cells to present self-peptides that facilitate both Treg development and clonal deletion.

In addition to TCR signals, IL2 plays a key role, as indicated by defective thymic Treg development and subsequent autoimmunity in mice lacking IL2, or its high-affinity receptor chain CD25 (also called IL2Rα; see Box 6) (Furtado et al., 2002; Malek et al., 2002). The relationship between TCR and IL2 signals was clarified by studies showing that strong TCR signals led to CD25 upregulation on CD4 SP thymocytes and that CD4 SP CD25^+^Foxp3^−^ cells could upregulate *FoxP3* upon intrathymic injection (Box 5) or in an in vitro culture in the presence of IL2 (Lio and Hsieh, 2008) (Fig. 3 A). Moreover, STAT5, which is downstream of IL2, bound to and activated the *Foxp3* gene (Burchill et al., 2008). Together these data supported a two-step model for Treg development with strong TCR signal conferring IL2 responsiveness and IL2 driving FoxP3 expression via STAT5. (Burchill et al., 2008; Lio and Hsieh, 2008) (Fig. 3 A).

Box 5 Because Treg development is most efficient at low clonal frequencies, experimental approaches have been devised to introduce small but trackable clonal Treg precursor populations into the thymus. First, intrathymic injection is an ultrasound-guided method of introducing small populations of TCR transgenic thymocytes into the thymus of a nontransgenic mouse (Georgiev et al., 2023). Second, mixed hematopoietic bone marrow chimeras are made when irradiated mice are reconstituted with a low frequency mixture (1:10 or less) of TCR transgenic and wild-type bone marrow. Intrathymic injection and mixed bone marrow chimeras are in vivo methods that result in detectable clonal Treg development (Bautista et al., 2009; Georgiev et al., 2023; Leung et al., 2009; Moran et al., 2011). Third, an ex vivo method using thymic tissue slices has also been used to study Treg development (Weist et al., 2015). When thymic tissue slices containing agonist ligands are overlaid with MHC-II specific TCR transgenic thymocytes, a low frequency (<1%) of transgenic thymocytes migrate into the slice and undergo a synchronized wave of Treg development over several days.

One striking feature of thymic Treg development is the impact of clonal competition. This was first noted when TCR transgenic mice expressing Treg-biased TCRs were generated, and surprisingly, little Treg development was observed. On the other hand, when the frequency of transgenic thymocytes was reduced (see Box 5), transgenic T cells could give rise to Tregs, with the proportion of Tregs inversely proportional to the frequency of transgenic thymocytes (e.g., clonal Treg precursors) in the thymus (Bautista et al., 2009; Leung et al., 2009). These data imply the existence of limiting niches that Treg precursors must access to develop into Tregs. Initially, it was suggested that the limiting factor for Treg development might be rare high affinity (agonist) self-peptides, and subsequent studies using a reporter for TCR signal strength (Nur77-GFP; Box 1) confirmed this suggestion. When a Treg-biased TCR was expressed by all thymocytes, reporter expression was comparable with conventional CD4 SP thymocytes. However, when the number of transgenic thymocytes was reduced to a frequency that allowed for Treg development, reporter expression rose, implying that efficient recognition of agonist ligands occurred only when the frequency of Treg precursors was low (Moran et al., 2011). Subsequent studies provided evidence that IL2 is also a limiting factor and that existing Tregs inhibit new Treg development by competing for limited quantities of IL2 (Thiault et al., 2015; Weist et al., 2015) (Fig. 3 B). In addition, studies using a thymic tissue slice model for Treg development (Weist et al., 2015) (Box 5) showed that very low levels of IL2 produced by antigen-bearing dendritic cell (DC) could effectively promote Treg development, implying that self-agonist ligands and IL2 were most effective when they were part of the same physical niche.

It is interesting to consider how competition for antigen and IL2 could function together to determine which Treg precursors develop into Tregs (Fig. 3 B). A Treg precursor that successfully competes for access to agonist ligand (perhaps because it arrived first to the niche) would likely be a better competitor for IL2 due to its greater upregulation of CD25 compared with a late-arriving Treg precursor. In addition, IL2 signaling through STAT5 further increases the expression of CD25 (Barthlott et al., 2005; Busse et al., 2010; Kim and Leonard, 2002), which would tend to give a further advantage to the front runners in the IL2 competition. TCR affinity for self-ligands may also influence the “fitness” of a Treg precursor for competing for niche access. Indeed, higher TCR affinity for self correlates with more efficient Treg development (Lee et al., 2012), perhaps in part due to stronger induction of CD25 and a greater ability to compete for IL2.

Another interesting twist to competition in the Treg niche comes from the observation that autoreactive CD4 T cells are a major source of IL2 in the thymus (Hemmers et al., 2019; Owen et al., 2018). It is likely that some Treg precursors that engage agonist ligands but fail to become Tregs due to insufficient IL2 likely give rise to autoreactive T cells. These autoreactive T cells may remain within the niche and produce IL2, thereby expanding the ability of that niche to promote the development of Treg with related antigen specificity (Fig. 3 C). This is in line with the “buddy hypothesis” (Hsieh et al., 2012; Klein et al., 2019), which posits that the thymus produces a balanced output of autoreactive T cells together with Tregs of related specificity that can hold the autoreactive T cells in check.

### Beyond IL2 and the two-step model

While IL2 plays a central role in thymic Treg development, other related cytokines can support Treg development and appear to favor distinct developmental pathways and TCR specificities. Most notably, the STAT5-activating cytokine IL15 (Box 6) is a significant player, with mutations that disrupt both IL2 and IL15 signaling leading to a much greater reduction in thymic Tregs than mutations of IL2 alone (Apert et al., 2022; Burchill et al., 2007, 2008; Fontenot et al., 2005; Lio and Hsieh, 2008; Soper et al., 2007; Vang et al., 2008). Moreover, in apparent contradiction to the two-step model, which posits that CD25 induction is required prior to *FoxP3* upregulation, the thymus contains a substantial population of developing Tregs that lack CD25 but express *FoxP3* (Marshall et al., 2014; Owen et al., 2019a). Interestingly, loss of IL15 signaling selectively impacts CD25^−^FoxP3^+^ thymic Tregs, whereas loss of IL2 signaling selectively impacts the CD25^+^FoxP3 population (Marshall et al., 2014). CD25^+^FoxP3^+^ thymocytes appear to have higher self-reactivity than CD25^−^FoxP3 thymocytes based on the expression of the Nur77-GFP TCR signaling reporter (Owen et al., 2019b) (Box 1). This difference may relate to the requirement for IL2-dependent Tregs to upregulate CD25 to compete effectively for limiting supplies of IL2, and the lack of such a requirement for CD25^−^ Tregs. Given that IL2 and IL15 are produced and/or presented by distinct cell types and are thus likely to be associated with distinct Treg niches (Fig. 3, C and D), it makes sense that the TCR repertoire of the Tregs that developed in the absence of IL2 is distinct from those that develop in the absence of IL15 (Owen et al., 2019b; Apert et al., 2022). Thus, instead of simply representing redundant cytokines, IL2 and IL15 drive the development of distinct sets of Tregs with specificity for different sets of self-antigens.

Box 6 IL2 receptor family

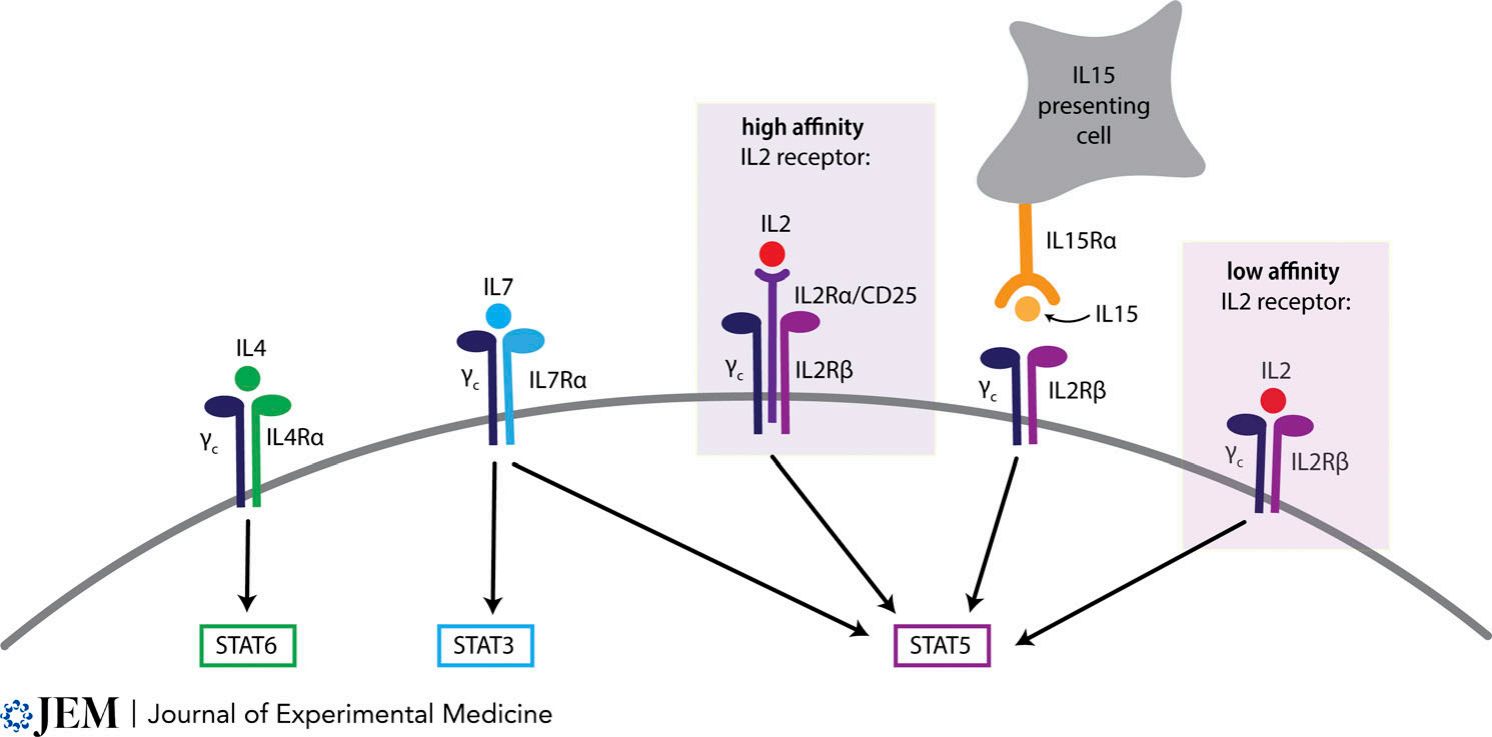

The common gamma chain (γ_c_) (also called CD132) pairs with several other chains to form heterodimeric receptors that bind different cytokines. IL7, IL4, and IL9 all signal through their respective receptor alpha chains paired with γ_c_ (Leonard et al., 2019). IL2 and IL15 share a heterodimeric receptor, IL2Rβ (also called CD122), that pairs with γ_c_ and signals through the signal transducer and transcription factor STAT5. In addition, the IL2 receptor can exist as a high-affinity trimeric form that includes the IL2Rα chain (also called CD25) (Lin and Leonard, 1997). Furthermore, IL15 is most efficient at signaling when it is trans-presented on IL15Rα expressed by an adjacent cell (Dubois et al., 2002).

Even when mice lack both IL2 and IL15, some thymic Treg development can still occur (Apert et al., 2022). In contrast, thymic Treg development is undetectable in mice lacking the common γ_c_ (Fontenot et al., 2005), implying that one or more of the other cytokines that signal through the common γ_c_ (see Box 6) may also contribute (Bayer et al., 2008; Chen et al., 2009; Mazzucchelli et al., 2008; Vang et al., 2008). It remains to be seen if, like IL15, other common γ_c_ cytokines may be important for particular subsets of Tregs, or if they simply function redundantly for any Treg precursor that may not have access to IL2. There is also evidence that the unrelated cytokine TGFβ, which signals through SMAD proteins and plays an important role in induced Treg development in the periphery (Chen, 2023), also contributes to thymic Treg development, although how that relates to the requirement for common γ_c_ and STAT5 remains unclear (Chen and Konkel, 2015; Liu et al., 2008). The potential impact of cytokines within different thymic niches will be discussed further below.

### Diverse medullary cell types serve as APCs for Treg development

The medulla contains a variety of APC populations that are specialized to facilitate both Treg development and clonal deletion. These include mTECs that use the transcriptional regulators Aire and Fezf2 to express large portions of the genome, making them a unique source of peptides that would normally be considered tissue-restricted (Anderson et al., 2002; Derbinski et al., 2001, 2005; Takaba et al., 2015). In addition, thymic DCs, which serve as the professional APCs of the thymus (Audiger et al., 2017), can present transferred mTEC-derived antigens (Gallegos and Bevan, 2004; Mouri et al., 2017; Perry et al., 2018) and can transport antigens from the periphery into the thymus (Bonasio et al., 2006; Zegarra-Ruiz et al., 2021). Both DC and mTECs can participate in Treg development (Anderson et al., 2005; Aschenbrenner et al., 2007; Bonasio et al., 2006; Gallegos and Bevan, 2004; Hinterberger et al., 2010; Liston et al., 2003; Malchow et al., 2013, 2016). In addition, TCR repertoire analyses from mice in which either mTECs or DCs lacked MHC-II revealed distinct roles for each APC type in selecting particular Treg specificities (Perry et al., 2014) (Box 4). More recently, thymic B cells have been implicated in tolerance to distinct sets of self-antigens (Afzali et al., 2024; Yamano et al., 2015).

In addition to displaying unique sets of self-peptides, other characteristics of medullary APC populations likely contribute to their ability to promote Treg development or clonal deletion. For example, mTECs can present IL15 but produce very little IL2, which may enhance their ability to promote Treg development via a CD25^−^ pathway (Cui et al., 2014; Marshall et al., 2014) (Fig. 3 D). In contrast, DCs may cultivate IL2-rich niches, both by making IL2 themselves (Weist et al., 2015; Apert et al., 2022) and by stimulating IL2 production from autoreactive CD4s (Hemmers et al., 2019; Klein et al., 2019; Owen et al., 2018) (Fig. 3 C). Thus, niches focused on DC may better support Treg development by a two-step CD25^+^ pathway. Medullary APCs also express varying levels of the costimulatory ligands CD80/86 that can boost TCR signals and influence whether a thymocyte undergoes Treg development or deletion (Coquet et al., 2013; Lancaster et al., 2019; Watanabe et al., 2020). In addition, thymic APC with greater ability to phagocytose thymocytes are particularly efficient at inducing negative selection (Kurd et al., 2019) and thus may favor clonal deletion over Treg development. A recently described thymic DC subset marked by CD301b^+^ plays an important, non-redundant role in medullary thymocyte deletion and also expresses TIM-4, a receptor for the phagocytic eat-me signal phosphatidyl serine (Breed et al., 2022).

### Multicellular niches for tolerance and Treg development

The diversity of the thymic medulla is not merely a direct consequence of tissue-restricted gene expression driven by AIRE and related transcriptional regulators such as Fezf2. Instead, tissue-restricted gene expression leads to the conversion of some mTECs into “mimetics” of distinct epithelial cell types that take on the morphology, cytokine signatures, and proteome of highly specialized cell types, such as microfold cells, hepatocytes, and Tuft cells. (Bornstein et al., 2018; Givony et al., 2023; Michelson et al., 2022; Miller et al., 2018). Differentiation into mimetic cells amplifies the expression of lineage-specific proteins, increasing the antigens available to present to thymocytes. Mimetic cells retain some of their mTEC character and can directly present tissue-specific antigens. In addition, thymic mimetic cells can recruit other nearby cells to allow for antigen transfer and to create complex niches for tolerance induction (Michelson and Mathis, 2022).

While the specific role of thymic mimetic cells in Treg development is currently unknown, early evidence suggests that they play an important role in tolerance induction. For example, thymic tuft cells, which were the first thymic mimetic cells to be identified (Bornstein et al., 2018; Miller et al., 2018), are found in clusters throughout the thymic medulla and influence their local tissue environment by producing IL25 and by inducing nearby NKT cells to produce IL4 (Fig. 3 E). Depletion of thymic tuft cells, by gene knockout of their transcriptional master regulator *Pou2f3*, leads to a loss of tolerance to IL25 (Miller et al., 2018). In addition, mTECs that resemble endocrine secretory cells, called EndoTECs, express a variety of peripheral secreted antigens (Givony et al., 2023). Depletion of EndoTECs by deletion of the gene encoding their transcriptional master regulator *Insm1* leads to the production of autoantibodies specific for endocrine-rich tissues, such as the stomach (Givony et al., 2023). Although it is unclear whether the self-tolerance induced by Tuft cells and EndoTECs is due to clonal deletion, thymic Treg production, or both, it will be of great interest to see how the composition of these niches impacts the mode of tolerance. For example, the type II cytokine environment surrounding tuft cells may favor clonal deletion by activating thymic CD301b^+^ thymic DCs that are adept at inducing negative selection (Breed et al., 2022).

Thymic B cells are part of another multicellular medullary niche for tolerance induction that includes mTECs and self-reactive CD4 SP thymocytes (Frommer and Waisman, 2010; Lu et al., 2015; Walters et al., 2014) (Fig. 3 F). Thymic B cells require both CD40 from auto-reactive CD4 SP thymocytes and IFN III from thymic epithelial cells and cannot efficiently select thymic Tregs in the absence of these signals (Martinez et al., 2023; Yamano et al., 2015). Mouse models with B cell deficiencies have reduced thymic Treg output (Lu et al., 2015; Martinez et al., 2023; Walters et al., 2014; Yamano et al., 2015), and model antigen expression by thymic B cells results in Treg development of thymocytes with antigen-specific TCRs (Frommer and Waisman, 2010). More recently, it was shown that thymic B cells are critical for tolerance to the autoantigen, Aquaporin 4 (AQP4). Interestingly, clonal deletion appeared to be the primary mechanism of tolerance in this model, although some Treg development was also observed (Afzali et al., 2024). There is also evidence of crosstalk between thymic B cells and thymic mimetic cells (Givony et al., 2023). Thymic microfold mimetics, which resemble intestinal M cells, coordinate with thymic DCs to induce nearby thymic B cells to class-switch to IgA. Microfold cell-deficient thymi have reduced IgA^+^ B cells, and it will be interesting to uncover the impact of this microfold-IgA B cell medullary niche on self-tolerance (Givony et al., 2023).

## Conclusions and future directions

The process by which αβT cells adjust to their self-reactivity takes a week or more, spans the cortex and medulla, shapes T cell fate decisions (CD8, CD4, and Tregs), and leads to functional tuning of TCR responses. While this review has highlighted recent progress in understanding this process, many questions remain. For example, what are the key molecular differences between conventional mature T cells of high (CD5^high^) versus low (CD5^low^) self-reactivity? Interestingly, the transcriptional differences between CD5^high^ versus low CD5^low^ T cells are modest (Fulton et al., 2015; Lutes et al., 2021; Rogers et al., 2021; Sood et al., 2021), suggesting that changes at the protein, metabolic, or epigenomic levels may also underlie the functional differences. Additionally, while TCR responsiveness dynamically varies as thymocytes undergo positive selection, the mechanistic basis of those changes is not well understood. Are different branches of the TCR signaling pathway amplified or dampened as thymocytes mature and give rise to CD4 or CD8 T cells, and how do these changes impact the ability of mature T cells to respond to TCR triggering during infections? Regarding Treg development in the medulla, it is becoming increasingly clear that the ability of newly generated CD4 SP to give rise to Tregs depends on the availability and composition of diverse medullary niches. The extraordinary diversity of medullary niches for tolerance induction is just beginning to be uncovered. The application of spatial transcriptomic and spatial proteomics of the thymus will undoubtedly be important in future studies of these remarkable structures and should help to shed light on how they promote Treg development or negative selection to achieve self-tolerance. Finally, while this review has focused on events in the young adult thymus, age-related changes in the thymic environment and T cell development are of great interest. In particular, the neonatal thymus generates distinct sets of conventional T cells (Rudd, 2020) and Tregs (Stadinski et al., 2019), and how unique environments and progenitor populations of the neonatal period impact tuning self-reactivity requires further investigation.
